# Effect of Lipid-Encapsulated Acacia Tannin Extract on Feed Intake, Nutrient Digestibility and Methane Emission in Sheep

**DOI:** 10.3390/ani9110863

**Published:** 2019-10-25

**Authors:** Festus Adeyemi Adejoro, Abubeker Hassen, Abiodun Mayowa Akanmu

**Affiliations:** Department of Animal and Wildlife Sciences, University of Pretoria, Private bag X20, Hatfield 0028, South Africa; Festus.adejoro@tuks.co.za (F.A.A.); Abiodun.akanmu@up.ac.za (A.M.A.)

**Keywords:** Acacia, Lipid-encapsulated tannin, methane emission, nitrogen balance, nutrient digestibility

## Abstract

**Simple Summary:**

Enteric methane is not just a contributor to anthropogenic greenhouse gas emission, it also represents a loss of potential feed energy. The use of *Acacia* tannin extracts as a dietary additive effectively reduced methane production while also modulating nitrogen loss. The reduction in methane output in sheep receiving the encapsulated-*Acacia* tannin extract (ATE), as compared to the crude-ATE diet, suggests the potential use of encapsulation to improve tannin inclusion in ruminant diets, by enabling the sustained release of the tannin to the rumen environment.

**Abstract:**

Tannins have become important phytochemicals in ruminant production, due to their wide range of biological activities. The use of a crude extract often comes with limitations, such as reduced feed intake and fibre digestibility, which could be overcome by the use of encapsulated tannin extract. In this study, four rumen-cannulated Merino wethers were used in a 4 × 4 Latin square design to determine the effect of encapsulating *Acacia mearnsii* tannin extract on intake, nutrient digestibility, and methane emission. The animals were placed on one of the following diets: control diet only, diet + silvafeed (Silvafeed ByPro, 10 g/kg feed), diet + *Acacia* tannin extract (ATE), 40 g/kg feed), and, diet + lipid-encapsulated-ATE (palm oil encapsulated ATE, 50 g/kg feed) in 4 cycles. Wethers were offered an Eragrotis and Lucerne hay-based total mixed ration diet above maintenance requirement with forage: concentrate ratio 50:50. Silvafeed, a commercial tannin additive, was used as a positive control. Nutrient intake was not different across the treatments, but nutrient digestibility was affected by dietary additives (*p* < 0.05). Compared to the control, and unlike the crude extract, encapsulated-ATE and silvafeed did not reduce dry matter, organic matter, and neutral detergent fibre digestibility. While the overall N-retention and total N-excretion (g/d) were not affected by dietary additives, ATE and encapsulated-ATE diets reduced urine-N excretion (g/d) and only a slight reduction was observed in silvafeed diet. The faecal-N proportion was highest in the ATE diet (388 g/kg N-intake), followed by encapsulated-ATE (317 g/kg), and silvafeed (267 g/kg), with the control diet having the lowest proportion (230 g/kg). The acetate:propionate (A:P) ratio reduced as a result of the inclusion of dietary additives with crude ATE and silvafeed having lower A:P ratio compared to the control diet. Methane production expressed in g/kg dry matter (DM) intake was reduced by 12%, 30% and 19% in the silvafeed, crude ATE and encapsulated-ATE diets, respectively (*p* < 0.05). The reduced methane production with higher neutral detergent fibre (NDF) digestibility in the encapsulated-ATE, compared to the crude-ATE, confirms that encapsulated-tannin can be used as an additive in ruminant diets.

## 1. Introduction

The use of plant secondary metabolites as natural alternatives to antibiotic growth promoters has continued to elicit great interest globally. Furthermore, supplementation with plant secondary metabolites is capable of improving livestock productivity while also reducing the impact of livestock production on the environment [1,2]. One type of plant secondary metabolite known for their wide application in animal production are condensed tannins, which are water-soluble polyphenolic compounds of high molecular weight with the ability to modulate rumen fermentation [3,4]. Past research has revealed that condensed tannins can bind with protein in feed, saliva, tissue, enzymes, and microbes. This results in reduced rumen protein degradability, and reduced urinary nitrogen loss [5,6]. Other applications of CTs include the control of bloat and intestinal parasites [7] in ruminants, as well as a reduction in enteric methane emissions [8,9,10]. Condensed tannins exhibit their anti-methanogenic activity directly by inhibiting the growth of methanogens through the tanning action of their functional proteins, resulting in bacteriostatic and bactericidal effects [11,12].

However, moderate to high concentrations of dietary CT, which proved consistently effective in methane reduction, may also significantly depress dry matter intake and nutrient digestibility depending on the concentration and biological activity of the tannin [3,13]. The astringency of tannins is particularly noted for the negative responses in feed intake observed in ruminants consuming high tannin concentrations [14,15]. Equally, a compromise on intake and nutrient digestibility, if not carefully balanced, may impose a serious challenge on production performance. Beyond the rumen, free CT may also re-bind to dietary protein, digestive enzymes, or membrane proteins of mucosal cells, thereby reducing nutrient absorption [16,17]. Recent studies have shown that encapsulation may be used to mask or reduce the negative effects associated with tannins and other bioactive compounds in the food or feed industry, with additional tendency to control the release of the active ingredient at the required site for optimum effectiveness [18,19,20].

Thus, encapsulation of tannin extract has the potential to mask the astringency by restricting the biological activity of tannins in the mouth and provide a sustained release in the rumen, guaranteeing the modulation of microbial fermentation, and, therefore, the tannin effect is limited to the rumen and post-rumen environment. According to Mamvura et al. [21], the productivity of bioactive compounds may be enhanced when they are fully available at the precise location where they are required such as in the rumen. It is hypothesised that encapsulated *Acacia* tannin extract (ATE) will confine the effect of tannin to the rumen, resulting in improved anti-methanogenic effect without an associated depression in feed intake. A gradual release of tannin in the rumen may equally ensure the minimal effect of tannin on nutrient absorption in the intestine.

The aim of this study, therefore, was to determine if encapsulating *Acacia mearnsii* tannin extract in a lipid matrix will favourably mask the bitter taste of tannin and possibly slow down its release in the rumen without reducing its antimethanogenic potency. By this, the anticipated utilisation of encapsulated CT extract in methane reduction can be achieved without the associated negative effects on dry matter intake, nutrient digestibility and N-balance, using South Africa Mutton Merino wethers as a model animal.

## 2. Materials and Methods

Animal management protocols were carried out following the University of Pretoria animal ethics committee guidelines as stipulated in the approval number EC061-14. The experiment was carried out at the small stock unit, Hatfield experimental farm, University of Pretoria (Pretoria, South Africa).

### 2.1. Micro-Encapsulation of Acacia Mearnsii Tannin Extract

*Acacia mearnsii* tannin extract was supplied by UCL Company (Pty) Ltd. Dalton, South Africa. The water-soluble powdered product was analysed for its total phenol, total tannin and condensed tannin concentrations, following the procedure described by Makkar [22]. The product contained 0.651 g/g DM total phenol, 0.58 g/g DM total tannin (as tannic acid equivalent) and 0.35 g/g DM of CT (as leucocyanidin equivalent). Encapsulated-ATE was prepared by the double-phase emulsion micro-encapsulation technique using palm oil as the lipid wall material [19].

### 2.2. Animals, Diets, and Experimental Design

The study was carried out from October 2017–February 2018. Four Merino wethers fitted with rumen cannulas (bodyweight, 75.8 ± 5.4 kg) were assigned to one of the four experimental diets in a 4 × 4 Latin square design. Diets contained Eragrostis and Lucerne hay as the main roughage fraction, with a concentrate to roughage ratio of 50:50. The animals received the diets in four different periods of 26 d each (i.e., 14 d of adaptation, 7 d of digestibility data collection and 5 d of methane emission measurement). At the commencement and termination of each period, animals were weighed and the average was used in calculating the average bodyweight of the individual animal. The four treatments were control diet (C), diet + silvafeed (Silvafeed ByPro, 10 g/kg), diet + crude-*Acacia* tannin extract (ATE; 40 g/kg), and diet + lipid-encapsulated *Acacia* tannin extract (encapsulated-ATE; 50 g/kg). Silvafeed ByPro^(R)^ is a commercial blend of Quebracho CT and Chestnut hydrolysable tannin, in powdered form, with total phenol concentration of 0.70 g/g (tannic acid equivalent) and 0.16 g/g CT (leucocyanidin equivalent). It was supplied as a single batch by Silvateam, S.p.a. (San Michele Mondovi, Italy). Based on the reports of Henke et al. [6], it was included at 10 g/kg DM as a positive control. The encapsulated-ATE contains about 0.80 g/g ATE from *Acacia mearnsii* as the active ingredient. Both ATE and encapsulated-ATE provided approximately 14 g/kg CT and selected based on the responses reported by Grainger et al. [23] where 9 g/kg and 18 g/kg CT from ATE was included in the diets of dairy cows.

### 2.3. Apparent Digestibility and N-Balance Measurement

Experimental animals were fed the total mixed ration diets *ad libitum*, and this was provided daily, in two instalments at 07:00 and 16:00 while clean water was equally provided *ad libitum*. The diet, which contained approximately 50% roughage from Eragrostis and Lucerne hay, was mixed with concentrate and vitamin-mineral premix using a vertical mixer to form a total mixed ration (Table 1). During each study period, tannin extract was added daily to the weighed ration, and hand-mixed thoroughly inside a wide plastic container, before offering it to the animals. Feed refusal was weighed once daily and recorded before the next feed was offered. Faeces was collected in faecal bags and urine was collected in metal trays with a funnel that emptied into a plastic bucket and total faecal excretion and urine output were recorded daily. The urine collection bucket contained 50 mL of sulphuric acid (10% v/v) (Merck Millipore KGaA, Darmstadt, Germany) to prevent N-volatilisation. Samples of diet, orts, faeces and urine (10%) were collected daily and stored at −20 °C. This was subsequently pooled across days, within each period and sub-sampled for analysis. A sub-sample of feed, orts and faeces were oven-dried at 105 °C for 18 h for dry matter determination, while a second sub-sample was dried at 55 °C for 48 h, milled to pass a 1 mm sieve, and stored for chemical analysis. At the end of each period, rumen fluid was sampled via the cannula opening and pH was determined immediately using a pH meter (HANNA HI-8424, Charlton Scientific, Oxfordshire, UK), thereafter rumen fluid samples were taken for the analysis of volatile fatty acids (VFAs) by preserving the rumen fluid samples in 25% orthophosphoric acid (5:1 v/v) (Merck Millipore KGaA, Darmstadt, Germany) and for ammonia-N by preserving samples in 0.5 M sulphuric acid (6:1 v/v).

### 2.4. Open-Circuit Respiratory Chamber and Methane Emission Measurement

The open-circuit respiratory chambers are made of steel frames and ultraviolet-resistant clear flexible polyvinyl chloride (PVC) sheet on every side. The features are similar to the one described by Storm et al. [24]. The lower section of one side of each chamber has an air space to allow air inflow while at the top opposite side, an opening was made to channel air through a pipe connected to an exhaust fan to force air out of the chamber in one direction through the creation of negative air pressure. Each chamber was pre-calibrated via a methane recovery test by releasing a known concentration (99.5%) and amount of methane gas into the chambers and sampling methane concentration of outflowing air as well as the airflow through the exit ducts. This calibration of chambers was done at the start and the end of each cycle of the methane emission measurement. These recovery percentages (78%–107%) were thereafter used as a correction factor to adjust the total volume of methane produced by each animal as they rotated through each chamber. During methane measurement, animals were rotated across the 4 chambers, every other day during each period, to minimise the effect of between-chamber variation on the final methane output.

While the animals were inside the open-circuit respiratory chamber, feed supply was kept constant, and orts were weighed daily. The four animals, representing each treatment, were rotated every 24 h across the 4 different chambers by adapting the animals to the chambers for one day and this was followed by 4 consecutive days of methane output measurement. Animals were fed their ration in one daily portion, water was provided *ad libitum* and the chamber was cleaned within 1.5 h while air sampling occurred for the remaining 22.5 h. Airflow within each chamber was monitored via a fixed hot wire anemometer probe, fitted with automatic data-loggers, and airspeed was recorded every 5 s, from which mean airflow through the pipe was estimated. A sub-sample of ambient air entering and leaving each chamber was continually withdrawn using an 8-channel peristaltic pump (Masterflex 77292-50 L/S, Cole-Palmer Instrument, Vernon Hills, IL, USA.) at 5 min intervals from each chamber. This was collected continuously via tubings into big deflatable Teflon bags fitted with a 3-way corkscrew for approximately 22.5 h period. Approximately 10 sub-samples of air were withdrawn from each chamber’s collection bag and ambient bag using gas-tight syringes and analysed by gas chromatography, (SRI 8610C BTU Gas Analyser GC System, SRI Instruments, Bad Honnef, Germany) to determine the concentration of methane in each chamber. The GC was equipped with a solenoid column packed with silica gel and a flame ionisation detector. During each period, the GC was pre-calibrated with analytical grade 100, 250, 500 and 1000 ppm methane in nitrogen gas (Portagas, Sam Houston, Pasadena, TX, USA.). Net methane concentration (emission of each animal) was calculated as the difference in methane concentration of air coming out of each chamber less ambient-air methane concentration. Total daily methane emission was therefore calculated by multiplying the total air volume extracted from each chamber in a day with the net methane concentration for each chamber. The average recovery percentage determined for each chamber was used as a correction factor to adjust the daily methane volume produced by the animal in each chamber.

### 2.5. Chemical Analyses

Samples of feed offered, orts and faeces were analysed following the standard procedure described in the Association of Official Agricultural Chemists (AOAC) Official Methods of Analysis [25] for dry matter (DM; ID 934.01), total ash (ID 942.05) and crude protein (ID 968.06). Neutral detergent fibre (NDF) was determined following the procedure described by Van Soest et al. [26] using the ANKOM technology filter bag technique with the addition of heat-stable alpha-amylase and sodium sulphite. The acid detergent fibre (ADF) was also analysed (non-sequentially) using the ANKOM Technology Corp. (Fairport, NY, USA) fibre analysis procedure. Both NDF and ADF are expressed exclusive of residual ash. Rumen fluid for ammonia nitrogen was analysed following the phenol-hypochlorite method as described by Broderick and Kang, [27] using a Specord 200 Analytik Jena UV-Spectrophotometer (Konrad-Zuse-Strasse, Germany). Samples for volatile fatty acids were centrifuged at 15,000× g for 10 min at 4 °C and the supernatant was subsequently filtered using 0.45 µm microspore filter. Thereafter, samples were injected into the gas chromatograph (Shimadzu GC-2010 Tracera; Shimadzu corp., Kyoto, Japan) with Barrier Ionisation Discharge (BID) detector and fitted with a 30 m Inert Cap Pure Wax column (df = 0.25 µm, I.D. = 0.25 mm). The operational conditions of the column included carrier gas flow, 6.1 mL/min; an initial temperature of 100 °C, injection port temperature of 250 °C, detector temperature of 280 °C; the helium gas flow at the detector was 50 mL/min.

### 2.6. Statistical Analyses

Apparent nutrient digestibility, nitrogen balance and methane production were analysed as a 4 × 4 Latin square design, using PROC MIXED procedure of SAS 9.4 (SAS Institute, Inc., Cary, NC, USA) with the following model:

Y*_ijk_* = *µ* + P*i* + A*_j_* + T*_k_* + e*_ijk_*,

where Y*_ijkl_* is the observation from animal (independent variable); *µ* is the overall mean; P*_i_* is the effect of period (*I* = 1, 2, 3, 4), A*_j_* is the effect of animal (*j* = 1, 2, 3, 4), T*_k_* is the effect of treatment/diet (*k* = 1, 2, 3, 4) and e*_ijk_* is the residual error effect. Each animal was considered as a random effect while period and treatment/diet as fixed effects. Diet means were compared using Tukey’s test and results reported as least square means and standard error of the means. Contrast comparison was done between ATE and encapsulated-ATE diets, to evaluate the effect of encapsulating ATE.

## 3. Results

The addition of ATE, encapsulated-ATE or silvafeed as additives did not affect dry matter (DM) intake of the animals when expressed in g/d or g/kg BW^0.75^/d (Table 2). Similarly, dietary treatments did not show any effect on daily organic matter (OM), crude protein (CP), neutral detergent fibre (NDF) and acid detergent fibre (ADF) intake of animal across the diets. The daily dry matter intake of wethers ranged from 1341–1479 g/animal/d.

Tannin additives generally reduced DM, OM, CP, NDF and ADF digestibility in the Merino wethers (*p* < 0.05). Compared to the control diet, while ATE diet was lower in DM, OM, NDF and ADF digestibility, values for encapsulated-ATE and silvafeed diets were not significantly different from the control diet. However, dietary inclusion of encapsulated-ATE reduced CP digestibility, although to a lesser extent than the ATE diet, when compared with the control diet (*p* < 0.05). Equally, encapsulation of ATE improved NDF (*p* < 0.05) digestibility and slightly improved ADF digestibility (*p* = 0.06) in the animals consuming the encapsulated ATE compared to those that received the crude ATE.

Overall N-intake (g/kg BW^0.75^/day), N-excretion (g/d) and N-retention (g/d) was not affected by dietary inclusion of additives (Table 3). Faecal-N excretion, g/d was increased (*p* < 0.05) by the inclusion of tannin additives with animals that consume ATE diet having higher faecal-N compared to the control, silvafeed and encapsulated-ATE diets. When expressed in g/kg N-intake, animals fed ATE produced highest faecal-N, followed by encapsulated-ATE, with both groups having higher faecal-N output compared to the control and silvafeed diets. Inclusion of additives generally affected urinary-N excretion, g/d (*p* < 0.05). But while urinary-N was unaffected by silvafeed diet, it was lower in the ATE and encapsulated-ATE diets. However, the proportion of urinary-N in total-N intake was not affected by dietary treatments.

The rumen fermentation parameters of sheep that received silvafeed, ATE or encapsulated-ATE are shown in Table 4. Rumen pH and ammonia concentration in the experimental animals were not affected by the inclusion of the various additives. There was equally no difference between animals that received the crude ATE and encapsulated-ATE diets in terms of pH and ammonia concentration. The total volatile fatty acid (VFA) concentration, as well as acetate, butyrate, iso-butyrate, iso-valerate and valerate proportions, were not affected by the diets. Propionate proportion was equally not affected by supplementation with control diet having was 19.4 mol/100 mol, while silvafeed, ATE and encapsulated-ATE diets produced 24.9, 27.9, and 21.2 mol/100 mol, respectively. Propionate proportion was lower in the encapsulated-ATE compared to the ATE diet (*p* <0.05). Acetate to propionate (A:P) ratio was influenced by the inclusion of the additives (*p* < 0.05). When compared with the control treatment, while encapsulated-ATE resulted in a slight decrease in A:P ratio, a significant decrease was observed in the silvafeed and ATE diets.

The in vivo methane (CH_4_) emission by sheep as influenced by the addition of silvafeed, ATE and encapsulated-ATE is shown in Table 5. Inclusion of tannin additives resulted in reduced methane output when expressed in g/d, g/kg BW^0.75^/d or in relation to DM (g/kg DM intake) and NDF intake (g/kg NDF intake) (*p* < 0.05). Compared to the control diet, inclusion of both ATE and encapsulated-ATE reduced (*p* < 0.05) daily methane output, g/d by 32% and 25%, respectively, while silvafeed diet resulted in a 20% reduction in methane. Equally, methane output in g/kg DM-intake showed that silvafeed reduced methane by 12%, while ATE and encapsulated-ATE diets reduced methane output by 30% and 19%, respectively. Nevertheless, when methane production is expressed in terms of g/kg DM-digested, the inclusion of additives did not affect methane production. There were no differences between ATE and encapsulated-ATE in terms of methane emissions.

## 4. Discussion

The inclusion of silvafeed, encapsulated-ATE and ATE did not reduce DM and OM intake when compared with the control diet. Reduced DM and OM intake associated with tannin supplementation have been reported in some studies as a limitation [14], while others have reported that tannin supplementation did not affect dry matter and nutrient intake [28,29]. In a study by Aboagye et al. [30], dry matter intake of beef steers was not affected by hydrolysable or a combination of hydrolysable and condensed tannin, at the inclusion level of 15 g/kg DM. Equally, up to 30 g/kg DM, feed intake was not affected by silvafeed supplementation in dairy cows [6]. The influence of tannin on nutrient intake may vary widely and depend on several factors such as the concentration of tannin in the diet, animal factor, biological characteristics of the tannin compound, effect of prolonged adaptation, rumen fermentation or diet characteristics [17,28,31]. The tendency for astringency to wane after an adaptation period, as previously observed by Landau et al. [32], may have occurred in this study. As the literature makes clear, one of the adaptive mechanisms to high dietary tannin by ruminants is the change in composition and volume of saliva secretion, with the possible tannin binding to salivary protein [13]. This mechanism is expected to sustain feed intake, rumination and reduce rumen retention time. In a related study, quebracho tannin consumption increased saliva total protein concentrations up to four-fold, in both sheep and goats [33]. Aside from the direct astringency of tannins resulting in the precipitation of salivary proteins [13], the reduced microbial activity resulting in declining nutrient digestion can trigger reduce feed intake in animals. However, Henke et al. [6], observed a reduced CP, NDF and ADF digestibility of silvafeed at 15 g/kg, but a reduction in feed intake was not observed up to 30 g/kg inclusion.

The reduced digestibility of DM, CP, OM, NDF and ADF recorded in the crude ATE and encapsulated ATE is related to the ability of tannin to bind to protein and fibre, thereby reducing their digestibility in the rumen or preventing microbial attachment to feed [5,10,23]. The lower inclusion level of silvafeed in this study may be responsible for the lack of depression in dry matter digestibility compared to the report by Henke et al. [6], where at 15 g/kg nutrient digestibility was reduced significantly. Compared to the crude ATE, a significantly higher CP and NDF digestibility in the lipid-encapsulated ATE diet may be an indication that more protein and fibre may have been degraded via microbial action as a result of the encapsulation due to a possible slow release of the tannin from the encapsulated-ATE. In a previous in vitro study, the rapidly fermentable fraction (‘a’), as well as rate of fermentation of the slowly fermentable fraction (‘c’) of a total mixed ration substrate, were higher when lipid-encapsulated tannin was included in the substrate instead of the crude tannin [19]. Similarly, in an in vivo study, an observed decrease in rumen degradability of CP was mainly associated with a significant reduction in its initial solubilisation (decrease in “a” value of digestion kinetics) [5]. Reduced salivation, slower passage rate as a result of slower solubility of encapsulated tannin [34] or decreased complexing with lignocellulose and feed protein, bacteria or enzymes [31] may have been responsible for the higher CP and NDF digestibility recorded in the encapsulated-ATE diet. Encapsulation may have ostensibly made tannin release slower in the rumen before the establishment of new linkages with protein and other feed nutrients as observed for fumaric acid in the rumen [20]. This mechanism for slow release of tannin may offer a huge potential in the administration of tannins and other phytochemicals in animal nutrition. High tannin concentrations can prevent microbial attachment to feed, impair fermentation and reduce nutrient and energy availability for rumen microbial growth [5,35]. Fibrolytic bacteria species in particular are known to be sensitive to tannins [36]. With encapsulation, microbial attachment to feed can be enhanced when tannin is slowly released from the lipid wall material while still ensuring the modulation of rumen nitrogen metabolism.

Higher faecal-N and lower urinary-N in ATE and encapsulated-ATE diets are consistent with previous reports on the effect of tannin on nitrogen partitioning [3,16]. Nevertheless, values for N-retention and total-N loss across the diets were not significantly different. This has equally been observed in a previous report [37]. Reduced nitrogen absorption in animals receiving tannin diets can cause a lack of differences in total N-retention. The absorption of nitrogen post-rumen is dependent on the reversibility of tannin-protein complexes, and this may not dissociate completely in the small intestine, or tannins may rebind to endogenous protein [15], thus increasing faecal-N loss. The extent of dissociation is dependent on the type of tannin-protein complex formed, and which is attributable to the structures of the tannin and proteins. Incomplete dissociation may, therefore, result in a lack of differences in the overall N-retention between control animals and those that received tannin additives. Despite the dietary treatments not affecting N-retention, a shift in N-excretion from urine to faeces is considered a more environmentally friendly form of nitrogen loss [37,38]. While faecal-N is in organic form, urine-N is largely in the form of urea that can be quickly hydrolysed to ammonia and nitrified to nitrate. Nitrate can leach into groundwater as a pollutant or can be converted to nitrous oxide, which is equally a potent greenhouse gas [39]. In contrast, the CT-protein complex in faeces will improve the quality of manure as a fertiliser, by ensuring that the nitrogen is slowly released into the soil due to the slow dissociation of CT-protein complex [39]. This would indirectly reduce the environmental impact of livestock production over a long-term period.

The lack of differences in some of the rumen fermentation parameters would be due to the small number of animals per treatment which limited the variability associated with diet effects. Although not significant, there was a tendency for reduced rumen ammonia concentrations in animals receiving tannin additives. Silvafeed, ATE, and encapsulated-ATE supplemented diets produced 11%, 30% and 26% lower rumen ammonia concentration, respectively, compared with the control diet. Significant reduction in rumen ammonia concentration is noted as a direct consequence of dietary tannin in the diet of ruminants [1,40], with ammonia being the end-product of ruminal microbial fermentation of proteins and non-protein nitrogen. Rumen pH and total volatile fatty acid (TVFA) concentration were not affected by the inclusion of silvafeed, ATE or encapsulated-ATE in the diets of the animals. This result is consistent with some reports on the effect of tannin extract on TVFA in sheep and cattle [10,41]. In the current study, TVFA and acetate production were not affected by tannin diets, and this was similar to the observation of Cieslak et al. [1]. On the other hand, propionate tended to be higher, resulting in a significantly reduced A:P ratio in the silvafeed and ATE diets. A decrease in A:P ratio was similarly observed by Molina-Botero et al. [42]. Lack of differences in TVFA and acetate may be an indication that overall, dietary additives did not significantly influence digesta passage rate or VFA absorption through the rumen wall [43]. While Carulla et al. [10] and Beauchemin et al. [28] observed a reduced A:P ratio in the rumen with supplementation of *Acacia* tannin and quebracho tannin respectively, Aboagye et al. [30] did not observe any change in A:P ratio in beef cattle supplemented with chestnut tannin or a combination of chestnut and quebracho. Compared to the crude ATE extract, A:P ratio was higher in the encapsulated-ATE animals, as a result of higher propionate proportion. The lower A:P ratio associated with tannin is usually due to a reduction in the activities of acetate forming bacteria which result in an increased propionate proportion [1]. The effect of tannins on the mixed rumen microbiome, particularly on the tannin-sensitive fibrolytic species may have been attenuated by encapsulation as reflected by improved NDF digestibility in the encapsulated-ATE compared to the crude-ATE diet. It has been noted that high condensed tannin and hydrolysable tannin could alter rumen biodiversity without affecting the overall microbial population structure [44].

The silvafeed, ATE and encapsulated-ATE diets reduced methane production, expressed in g/kg DM intake, by 12%, 30% and 19% respectively, but methane production per unit of DM digested was not significantly different across the diets. Nevertheless, the methane-reducing potential of *Acacia* tannin and other tannin additives has been noted in previous studies [10,23]. The methane suppressing effects of tannins relate to a combination of direct toxicity on the methanogenic archaea, reduced fibre degradation or reduced OM digestibility [15,28]. Even though quebracho CT supplementation in dairy cows up to 20 g/kg did not result in a significant reduction in methane as reported by Beauchemin et al. [28], nevertheless, silvafeed produced slightly lower methane than the control diet. The combination of chestnut with quebracho in silvafeed may have played an additional role in methane suppression as opposed to quebracho tannin alone. According to Jayanegara et al. [45], hydrolysable tannins had a greater effect in reducing enteric methane emission than condensed tannin. The crude ATE and encapsulated-ATE diets produced comparable methane output (17.2 vs 20.0 g/kg DM intake), with both diets producing significantly lower methane than the control diet. The result of this study is an indication that the encapsulation of ATE did not reduce its efficacy in methane suppression. The commercial use of extracts such as lipid-encapsulated ATE could have importance in mitigation of greenhouse gas from agriculture. However, farmer acceptance for such a product would largely depend on the benefits for sustainable animal production being able to exceed the cost of inclusion of such a dietary additive. The encapsulated-ATE showed potential to enhance the utilisation of ATE as evidenced by higher NDF digestibility with a significant reduction in methane production. As noted by Waghorn [16], while CT protects proteins from excessive ruminal degradation and reduces enteric methane, it can also inhibit the absorption of the nitrogen in the intestine or reduce fibre digestion. The use of tannin products, such as the encapsulated-ATE, in meeting this delicate balance, would, therefore, require further studies.

## 5. Conclusions

Silvafeed, crude-ATE and encapsulated-ATE in the diet of sheep reduced CP digestibility, while crude-ATE and encapsulated-ATE increased faecal-N excretion at the expense of urinary-N. Daily methane production was significantly reduced by both crude-ATE and encapsulated-ATE, but methane produced per unit of DM digested was not significantly affected. Encapsulated-ATE reduced methane production with higher NDF digestibility when compared to the crude-ATE.

## Figures and Tables

**Table 1:** Gross composition of ingredient and chemical composition of experimental diet.

Ingredients ^1^ (g/kg of DM)	Control Diet	Silvafeed	Tannin	Encapsulated-Tannin
*Eragrostis curvula* hay	300	297	287.9	285
Alfalfa hay	200	198	192	190
Hominy chop	140	138.6	134	133
Wheat bran	90	89.2	86.4	85.5
Maize germ meal	190	188	182	181
Urea	10	9.9	9.60	9.50
Molasses	60	59.4	57.6	57.0
Mineral and vitamin premix ^2^	5	4.95	4.80	4.75
Calcium carbonate	5	4.95	4.80	4.75
Silvafeed	0	10	0	0
Tannin	0	0	40	0
Encapsulated-tannin extract	0	0	0	50.0
Total	1000	1000	1000	1000
Analysed chemical composition				
Dry matter, (g/kg)	908	910	910	909
Organic matter, g/kg DM	922	921	939	930
CP, g/kg DM	162	152	146	150
NDF, g/kg DM	374	387	391	395
ADF, g/kg DM	211	211	220	219

^1^ All ingredients was provided as a total mixed ration. DM, dry matter; CP, crude protein; NDF, neutral detergent fibre; ADF, acid detergent fibre. ^2^ providing per kg: Zn, 25 g; Mn, 15 g; Se, 0.5 g; Co, 0.3 g, mg, 30 g, Fe, 15 g, Na, 500 mg and Cl, 280 mg; vit A, 2500 IU; vit D, 400mg; vit E, 3.5IU.

**Table 2:** Feed intake and apparent nutrient digestibility in Merino wethers fed total mixed ration containing crude or lipid-encapsulated *Acacia* tannin extract (ATE) and Silvafeed.

Parameter ^1^	Control	Silvafeed	ATE ^2^	Encapsulated ATE	SEM ^3^	*p*-Values ^4^	
						Diet	ATE vs E-ATE
DMI, g/d	1479	1386	1341	1359	71.6	0.57	0.86
DMI, g/kg BW^0.75^/d	57.3	53.8	51.9	52.7	2.61	0.53	0.84
OM, g/d	1358	1287	1228	1279	2.46	0.49	0.60
OM, g/kg BW^0.75^/d	52.6	49.9	47.6	49.6	2.42	0.27	0.57
CP, g/d	244	208	203	195	11.0	0.19	0.62
NDF, g/d	543	538	517	547	27.9	0.55	0.48
ADF, g/d	307	304	284	307	15.4	0.50	0.32
Apparent nutrient digestibility, %							
DM	63.2 ^a^	64.3 ^a^	50.4 ^b^	59.1 ^a^	1.52	0.01	0.01
Organic matter	64.2 ^a^	65.6 ^a^	51.2 ^b^	60.8 ^a^	1.53	0.01	0.01
Crude protein	77.1 ^a^	73.3 ^a^	61.2 ^c^	68.3 ^b^	1.30	0.01	0.01
NDF	38.6 ^a^	45.0 ^a^	24.0 ^b^	43.0 ^a^	2.53	0.01	˂0.01
ADF	37.7 ^a^	43.8 ^a^	21.8 ^b^	34.6 ^ab^	2.99	0.01	0.06

^1^ DMI, dry matter intake; BW^0.75^, metabolic body weight; OM, organic matter; CP, crude protein; NDF, neutral detergent fibre; ADF, acid detergent fibre. ^2^ ATE, *Acacia* tannin extract. ^3^ SEM, standard error of means. ^4^ ATE vs E-ATE, contrast comparison between *Acacia* tannin and Encapsulated-ATE diets. LSMeans with different superscript across a row are significantly different at *p* < 0.05.

**Table 3:** Nitrogen intake, excretion and retention in Merino wethers fed total mixed ration containing crude or lipid-encapsulated *Acacia* tannin extract (ATE) and Silvafeed.

Parameter	Control	Silvafeed	ATE ^1^	Encapsulated ATE	SEM ^2^	*p*-Values ^3^	
						Diet	ATE vs E-ATE
N-excretion, g/d	28.8	24.0	22.9	22.8	1.90	0.48	0.97
Faecal-N, g/d	8.99 ^b^	8.90 ^b^	12.7 ^a^	9.91 ^b^	0.76	0.04	0.04
Urinary-N, g/d	19.8 ^a^	15.1 ^ab^	10.3 ^b^	12.9 ^b^	1.47	0.02	0.25
Retained-N, g/d	10.2	9.3	9.6	8.4	1.43	0.84	0.57
N partitioning (% of N-intake)							
Faecal-N proportion, g/kg N-intake	230 ^c^	267 ^c^	388 ^a^	317 ^b^	13.0	0.01	0.01
Urinary-N proportion, g/kg N-intake	512	455	314	413	40.7	0.22	0.14
Retained-N proportion, g/kg N-intake	259	279	298	270	41.7	0.98	0.66

^1^ ATE, *Acacia* tannin extract; ^2^ SEM, standard error of means. ^3^ ATE vs E-ATE, contrast comparison between *Acacia* tannin and Encapsulated-ATE diets. LSMeans with different superscript across a row are significantly different at *p* < 0.05.

**Table 4:** Ruminal fermentation characteristics in Merino wethers fed total mixed ration containing crude extracts or lipid-encapsulated *Acacia* tannin extract (ATE) and silvafeed.

Parameter	Control	Silvafeed	ATE ^2^	Encapsulated ATE	SEM ^3^	*p*-Values ^4^	
						Diet	ATE vs E-ATE
pH	6.53	6.56	6.66	6.56	0.04	0.37	0.90
NH_3_-N, mg/dL	19.4	17.3	13.5	14.3	2.17	0.09	0.80
Total volatile fatty acid (VFA), mmol/L	96.4	104.8	83.6	91.3	7.51	0.33	0.50
VFA molar proportion, mol/100 mol							
Acetate	57.2	56.0	54.5	55.8	2.20	0.58	0.69
Propionate	19.4	24.9	27.9	21.2	1.43	0.07	0.02
Butyrate	19.6	15.3	13.0	18.5	2.30	0.62	0.14
Branched-chain VFA ^1^	2.48	2.32	2.84	2.90	0.40	0.68	0.18
Valerate	1.42	1.76	1.98	1.92	0.13	0.24	0.75
Acetate: propionate ratio	2.98 ^a^	2.32 ^bc^	1.99 ^c^	2.69 ^ab^	0.19	0.04	0.04

^1^ Iso-butyrate + Iso-valerate. ^2^ ATE, *Acacia* tannin extract. ^3^ SEM, standard error of means ^4^ ATE vs E-ATE, contrast comparison between *Acacia* tannin and Encapsulated-ATE diets. LSMeans with different superscript across a row are significantly different at *p* < 0.05.

**Table 5:** Methane (CH_4_) emissions in Merino wethers fed a total mixed ration containing crude extract or lipid-encapsulated *Acacia* tannin.

Parameter	Control	Silvafeed	ATE ^1^	Encapsulated-ATE	SEM ^2^	*p*-Values ^3^	
						Diet	ATE vs E-ATE
CH_4_, g/d	35.5 ^a^	28.5 ^ab^	24.2 ^b^	26.7 ^b^	2.07	0.04	0.43
CH_4_, g/kg BW^0.75^/d	1.37 ^a^	1.10 ^b^	0.94 ^b^	1.05 ^b^	0.08	0.03	0.37
CH_4_, g/kg DM intake	24.6 ^a^	21.7 ^ab^	17.2 ^c^	20.0 ^bc^	1.14	0.02	0.13
CH_4_, g/kg NDF-intake	65.8 ^a^	54.8 ^b^	44.4 ^c^	51.3 ^bc^	2.93	0.01	0.15
CH_4_, g/kg DM-digested	38.7	32.3	36.3	33.6	3.39	0.58	0.59

^1^ ATE, *Acacia* tannin extract. ^2^ SEM, standard error of means. ^3^ ATE vs E-ATE, contrast comparison between *Acacia* tannin and Encapsulated-ATE diets. LSMeans with different superscript across a row are significantly different at *p* < 0.05.
